# Health Education Advanced Leadership for Zimbabwe (Healz): Developing the Infrastructure to Support Curriculum Reform

**DOI:** 10.29024/aogh.19

**Published:** 2018-04-30

**Authors:** Eva M. Aagaard, Susan C. Connors, Amelia Challender, Jonathan Gandari, Kusum Nathoo, Margaret Borok, Midion Chidzonga, Michele Barry, Thomas Campbell, James Hakim

**Affiliations:** 1 Department of Medicine, Division of Medical Education, Washington University School of Medicine, St Louis, MO, US; 2 The Evaluation Center, School of Education and Human Development, University of Colorado Denver, Denver, CO, US; 3 NECTAR MEPI Program, University of Zimbabwe College of Health Sciences, Harare, ZW; 4 Department of Pediatrics, University of Zimbabwe College of Health Sciences, Harare, ZW; 5 Department of Medicine, University of Zimbabwe College of Health Sciences, Harare, ZW; 6 Department of Dentistry, University of Zimbabwe College of Health Sciences, Harare, ZW; 7 Department of Medicine, Division of General Internal Medicine, Stanford University, Palo Alto, CA, US; 8 Department of Medicine, Division of Infectious Diseases, University of Colorado School of Medicine, Aurora, CO, US

## Abstract

An economic crisis in Zimbabwe from 1999–2009 resulted in a shortage of faculty at the University of Zimbabwe College of Health Sciences (UZCHS) and declining enrollment and graduation rates. To improve proficiency and retention of graduates, the college sought to develop a competency-based curriculum using evidence-based educational methodologies. Achievement of this goal required a cadre of highly qualified educators to lead the curriculum review and innovation processes. The Health Education Advanced Leadership for Zimbabwe (HEALZ) program was established in 2012 to rapidly develop the needed faculty leadership. HEALZ is a one-year program of rigorous coursework delivered face-to-face in three intensive one-week sessions. Between sessions, scholars engage with mentors to conduct a needs assessment and to develop, implement, and evaluate a competency-based curriculum. Forty scholars completed training from 2012–15. All participants reported they were satisfied or extremely satisfied with the training after each week. Pre-post surveys identified significant knowledge gains in all key content domains. The program garnered significant organizational support. Scholars showed significant variation in progress toward implementing and evaluating their curricula as well as the quality of the work demonstrated by program end. Interviews of scholars and UZCHS leaders revealed important impacts of the program on the quality and culture of medical education at the college.

## Background

An economic crisis in Zimbabwe from 1999–2009 had far-reaching negative effects on medical education at the country’s primary health professions school, University of Zimbabwe College of Health Sciences (UZCHS). During this period, many UZCHS faculty emigrated to pursue careers outside Zimbabwe, and it was difficult to recruit and retain new junior faculty. Support for medical education deteriorated, and there was minimal investment in new infrastructure.

By 2010, 192 of 314 (61%) faculty positions at UZCHS were unfilled, and UZCHS was forced to decrease student enrollment by 49% (from 204 in 2006 to 105 in 2009) despite an abundance of qualified applicants [1]. Among other factors, these circumstances precipitated an alarming drop in medical school graduation rates, which exacerbated the shortage of qualified medical practitioners such that, by 2009, only 33% of doctor posts in the government healthcare system were filled nationwide [2].

The Novel Education Clinical Trainees and Researchers (NECTAR) grant funded by the Medical Education Partner Initiative (MEPI) was developed to disrupt the cycle of declining medical practitioner capacity in Zimbabwe. NECTAR was a consortium of faculty with a long history of strong and productive collaborations in education and research that included UZCHS, Stanford University, University of Colorado School of Medicine (UCSOM), and The Evaluation Center at the University of Colorado Denver (UCD). The goals of NECTAR were to (1) increase the number and proficiency of UZCHS graduates, (2) improve retention of UZCHS graduates in Zimbabwe, and (3) transform the UZCHS academic environment, creating new and sustainable educational and clinical partnerships and increasing research opportunities.

To improve the proficiency of graduates, NECTAR sought to develop a competency-based curriculum at UZCHS. Initially, we aimed to develop this curriculum with a focus on HIV, TB, and malaria [3,4]. Curriculum revision was subsequently expanded to comprehensively revise the entire undergraduate health professions curriculum at UZCHS.

Needs assessments performed by UCD and NECTAR evaluation teams indicated that investments in faculty leadership development were needed for successful implementation of the curriculum revisions. In surveys of UZCHS faculty, 100% agreed or strongly agreed that continued exposure to faculty development was necessary for their personal professional growth. Evaluation results also indicated a need for more in-depth training in curriculum development, educational scholarship, and change management. UZCHS faculty felt curriculum change was the responsibility of all faculty members and that a majority would need training to participate meaningfully in this change. UZCHS leaders believed the training should occur in person and in country, rather than occurring online or abroad, because of ongoing issues with internet bandwidth, faculty shortage, and limited funding. Based on these needs, a program for advanced training in education leadership was developed. In this paper we describe the development and evaluation of the Health Education Advanced Leadership for Zimbabwe (HEALZ).

## Intervention

Our working group (faculty from UZCHS, UCSOM, and UCD) designed the HEALZ Program in 2012. The goal was to rapidly develop a cadre of highly qualified medical educators to lead the curriculum review and innovation processes. Specifically, the aim was to enhance the educational capacity of UZCHS by developing skills in curriculum development, program evaluation, and educational leadership for faculty interested in pursuing advanced training in medical education. In addition, we sought to build a cohesive community of successful health professions educators. To achieve these goals, we designed a one-year program of rigorous coursework delivered face-to-face in three intensive one-week sessions Table 1. Between sessions, HEALZ scholars were expected to engage with mentors to conduct a needs assessment and to develop, implement, and evaluate a competency-based curriculum.

**Table 1:** HEALZ Content.

Week	Topics

1	Introduction to HEALZ, teamwork, and group expectations Being an effective mentee Principles of competency-based curriculum development and evaluation Developing a needs assessment Needs assessment methodologies: quantitative and qualitative methods
2	Data Analysis: quantitative and qualitative methodology Introduction to learning and pedagogy Writing goals and objectives Choosing educational strategies Learner assessment strategies Curriculum and program evaluation
3	Leading from personal strengths, understanding others Negotiating conflict Evaluating curriculum Managing change Giving and receiving feedback

The HEALZ curriculum was designed to be taught using a combination of experiential and small group strategies. The curriculum is spiral and iterative, and it incorporates both constructivist and social learning theories [5]. Content is based on the curriculum development training of Kern et al. [6]. During week one, scholars learn how to conduct a needs assessment (including a comprehensive literature review and investigation of existing curriculum) and about the basics of quantitative and qualitative research methods. Between weeks one and two, scholars develop and implement a needs assessment in their area of curricular interest. During week two, scholars learn skills to analyze needs assessment data and to develop their curriculum. We include lessons on developing competencies, goals, and objectives; on using educational strategies and learner assessments; and on program evaluation. Between weeks two and three, scholars analyze their needs assessment data and use it to develop their draft curriculum. During week three, we focus on project implementation, leadership skills, and change management (Appendix 1, available at https://evaaagaard1206.wixsite.com/website). Scholars are then expected to implement their curriculum through appropriate processes within the health sciences campus and their respective departments and assess its effectiveness and acceptability.

In addition, HEALZ includes activities designed to develop peer-mentoring relationships between scholars through daily small group interaction and team building exercises [7,8]. During these activities, scholars identify individual strengths, as well as mechanisms to call upon each other and their broader community for help in moving their goals forward. To provide ongoing support, we assign scholars a mentoring team consisting of both a local and distance mentor who provide content and methodology expertise [9]. Works in progress are presented at the beginning of each face-to face weekly meeting. Final projects are presented at an annual poster session and graduation ceremony.

## Evaluation Methods

As one indicator of the implementation of HEALZ, we documented participation and completion rates.

To measure the value of the program, we adapted Guskey’s five-level model of evaluation of professional development to collect data on (1) participants’ reactions, (2) perceptions of learning, (3) evidence of organizational support, (4) use of new knowledge and skills, and (5) evidence of impact on medical education in this setting [10].

To assess participants’ reactions and learning, we distributed exit surveys following each training week. Participants rated their overall satisfaction on a 5-point scale (1 = “not at all satisfied,” 5 = “extremely satisfied”). To collect data on participant learning, we asked scholars to self-assess their knowledge and skills in key content areas. Using a 5-point scale (1 = “No knowledge,” 2 = “Novice,” 3 = “Some knowledge,” 4 = “Knowledgeable,” 5 = “Expert”), participants rated their competence both retrospectively (“before this workshop”) and post-workshop (“now”). We combined results for cohorts One and Two for each of the three weeks. We analyzed differences in the knowledge self-ratings using Wilcoxon signed-ranks tests and calculated effect sizes. Exit surveys included open response questions to collect additional reactions from participants.

In 2013, independent evaluation team members who were not otherwise involved with HEALZ conducted interviews with scholars (n = 5) to collect further information about participants’ reactions, use of new knowledge, organizational support, and program impact. In 2014, interviews with scholars (n = 10) were conducted again to further examine the program’s impact on individuals. Interviews were audio recorded and transcribed. All interviews were analyzed using a grounded theory approach. Results were triangulated with other evaluation data.

To provide the scholars with feedback on their progress, we developed rubrics for scoring the curriculum project. The curriculum summary rubric (Appendix 2, available at https://evaaagaard1206.wixsite.com/website) includes a 4-point scale (1 = “Poor,” 4 = “Excellent”) in eight categories designed to assess scholars’ preliminary curriculum descriptions. HEALZ faculty other than an individual’s mentors reviewed each curriculum summary and shared scores and comments with the scholars. Results were analyzed using descriptive statistics.

Indicators of success at each of the five levels of evaluation of professional development are summarized below.

## Results

### Participation and Completion

Of 59 applicants, a total of 42 individuals were accepted into the HEALZ Program over a 3-year period (Table 2). Scholars in the first three cohorts represented 20 of the 23 departments within UZCHS (87%), a partner university in Zimbabwe (National University of Science and Technology), and the Zimbabwean Ministry of Health. Ninety-five percent of scholars completed the program.

**Table 2:** HEALZ Participation and Completion.

Cohort	# Applicants	# Scholars selected	# Graduates	# Mentors

One	21	14*	14 (100%)	11
Two	20	14	14 (100%)	42
Three	18	14	12 (86%)	38

*Plus 2 committee members who audited the program.

### Participants’ Reactions

All respondents (n = 41) in cohorts One through Three reported they were “satisfied” or “extremely satisfied” with their professional development after each training week. The satisfaction increased over time with 87% of respondents reporting they were “extremely satisfied” following cohort One’s third week. All respondents also indicated they were confident in their ability to complete their projects and reported they planned to use their learning in other aspects of their professional work. Responses were consistent across cohorts.

### Participants’ Learning

We found statistically significant differences between the pre and post scores in all HEALZ content areas; respondents as a group believed they increased in knowledge and skills in key content. While some individuals reported no change in some areas, the majority of respondents reported increased competence in all areas (Table 3). Effect sizes were large.

**Table 3:** Summary of Changes in Content Knowledge.

Module	Key content	N	Number of Survey Respondents Reporting			Z	Effect size *r*
			Increase	Decrease	No Change		

One	Principles of curriculum development	39	34	0	5	5.19*	.83
	Conducting a curriculum needs assessment	39	38	0	1	5.47*	.88
	Preparing quality surveys	39	34	0	5	5.28*	.85
	Conducting quality interviews	40	34	0	6	5.35*	.85

Two	Analyzing quantitative data	36	23	1	12	4.26*	.71
	Analyzing qualitative data	35	30	0	5	4.90*	.72
	Writing goals and objectives	34	22	0	12	4.28*	.73
	Choosing educational methods	36	32	0	4	5.10*	.85
	Assessing learners	36	24	0	12	4.67*	.78
	Developing plan for curriculum and evaluation	35	31	0	4	4.98*	.84

Three	Evaluating a curriculum project	42	33	1	8	5.03*	.78
	Writing about curriculum development for publication	42	35	2	5	5.14*	.79

* p < .001.

In addition, survey respondents also reported gaining competence in leadership and interpersonal skills, such as enhanced communication and improved interactions with colleagues in other disciplines.

### Organizational Support

HEALZ benefitted from early and active support from the UZCHS dean who participated in program design and recruitment and selection of scholars. This support was essential to the program’s successful inauguration. Support for the HEALZ program was also evident at the broader university level. In October 2013, the vice chancellor of the University of Zimbabwe delivered the keynote address at the first HEALZ graduation, an important marker of institutional support. In February 2013, he also established a Department of Health Professions Education within the university structure, which will oversee future faculty development efforts and serve as an academic home for programs like HEALZ. Representatives of the chancellor presented at the graduation ceremonies for the two subsequent cohorts as well.

HEALZ graduates co-facilitated the program beginning with cohort Three and assumed full responsibility for facilitating all sessions for cohort Four, as further evidence of organizational support and capacity.

Despite the institutional support described above, scholars also identified gaps. Specifically, interviewees reported a need for protected time to engage in coursework and project development, for funding to support project development, and for support in obtaining human subjects approval. Interviewees requested guidance from university leaders to support the implementation of their curricula.

### Participants’ Use of New Knowledge and Skills

HEALZ scholars provided evidence of their ability to apply new knowledge by preparing a curriculum summary, a written description of their project to facilitate implementation and to prepare the scholar for publishing their curricular work. The ratings of the projects for cohorts Two and Three show the areas of relative strength and weakness across projects (Table 4). For both cohorts, the rubric category with the highest average rating was “Quality of stated program goals and objectives,” although “Important/relevant to student learning” had the same average for cohort Three. Cohort Three had higher mean ratings than cohort Two on all rubric items except for one, “Pedagogically sound,” which had cohort Three’s lowest average rating. Cohort Two’s lowest average rating was for “Evidence-based.”

**Table 4:** Cohort Two and Three – Curriculum Summary Rating Averages.

Rubric Category	Average Rating (1–4)	
	Cohort 2	Cohort 3

Evidence-based	2.0	3.4
Important/relevant to student learning	2.33	3.7
Important/relevant to institutional goals, status, resources	2.67	3.5
Quality of stated program goals & objectives	3.33	3.7
Pedagogically sound	2.89	2.7
Educational strategies	2.67	3.1
Appropriate learner assessment	2.33	3.0
Program evaluation	2.44	3.3

In interviews, scholars reported they were applying their knowledge and skills in their role as medical educators. For example, one scholar said, “I changed my course curriculum, and it is now team-based learning. I cover it in an amazingly short period of time, and the students benefit more.” Another scholar explained:

In my daily work, like teaching, almost everything that I learned … I want to apply it in a deliberate sense. Maybe previously I did things with no background and with no real understanding of what I was doing. For example, when I am preparing lectures or when I am giving the lectures, I feel I am qualified to actually assess myself and I can identify where I think I could do better.

### Impact on Medical Education

As of April 2016, 17 of the 34 curriculum projects (Table 5) developed by HEALZ scholars were implemented; 13 projects were in development with plans to implement. For example, a scholar from cohort One developed a forensic psychiatry curriculum for post-graduate psychiatry trainees. The curriculum trained participants to assess and manage psychiatric conditions as related to criminal and civil legal issues and to develop skills in writing court reports so as to facilitate processing. Two years after the curriculum implementation, psychiatrists participating in forensic court evaluation increased from one to three (of 11 total psychiatrists in the country). Preliminary data suggest reduction in processing time for inmates with psychiatric co-morbidities. In cohort Two, a needs assessment revealed a critical curriculum gap in neonatology, with limited lecture-based content and no consistent clinical exposure. A competency-based curriculum consisting of didactic and experiential curriculum was implemented to fill the identified gap. This curriculum continues within the pediatric clerkship with support from faculty in pediatrics and surgery. Curriculum outcomes are in process.

**Table 5:** HEALZ Scholars’ Curricula Topics.

Cohort One	Cohort Two	Cohort Three

Physiology	Community preventive dentistry	Cardiovascular skills
Neonatology	Communication skills	Cerebral palsy caregiver training
Occupational safety and health	Community occupational therapy	Clinical supervisor training
Forensic psychiatry	Biostatistics	Community caregivers’ mental health
Genetics	Cardiac life support	Dentistry patient safety
Point of care tests	Ethical professionalism	Maternity patient safety
Professionalism and ethics	Hypertensive disorders of pregnancy	Pharmacology
Minimal access surgery	Child/adolescent mental health	Researcher skills
Stroke patient caregivers training	Gastroenterology	Upper GI endoscopy
Reproductive health/disease	Infection prevention/control	Urology
Rural field experiences	Neonatology	
	Neuroscience	
	Ophthalmology	

In interviews, scholars and UZCHS leaders reported HEALZ had contributed to the quality of medical education at the college. One specific theme of improvement was that HEALZ participation had increased interaction across departments. One scholar explained, “I was able to interact with medical people, with laboratory people, under one roof, over a long period of time. From there, we developed relationships that will last a lifetime because I can actually communicate with them without barriers.”

Another impact described by scholars was a shift in the culture within the college to a more student-centered approach. One scholar said, “I think one of the biggest things that I have learned and I think my colleagues have also learned is to appreciate the role of the students in their learning experience.” Scholars explained this shift was evident in the increased use of interactive teaching methods, the dissemination of explicit objectives, and the greater use of valid and fair assessment methods.

Other impacts on medical education noted by scholars were the renewed commitment to improving the curriculum both within and across departments and the implementation of new teaching methods. While HEALZ was not intentionally designed to improve teaching skills, scholars reported they learned new skills by adopting teaching methods used in the HEALZ program. One scholar described this process:

Observing teaching methods … I actually translated that into my teaching. … That was like a hidden curriculum for me because I used some of the methods that she was using, and I just copied it, and it is tremendous in terms of the impact it has made to my students.

UZCHS leaders also expressed the belief that HEALZ scholars were important assets for the curriculum review process both at the department and college level. One interviewee summarized the high expectations for the role of the HEALZ scholars stating, “We can now ensure that each department is equipped with resource persons with skills and knowledge for curriculum design, innovation, evaluation, teaching methods, and teaching assessment methods.”

## Discussion

Our program evaluation suggested very high levels of satisfaction and self-perceived gains in knowledge and skills. In addition, the program has garnered significant organizational support that has continued even after the completion of MEPI funding. Early on, scholars showed wide variation in progress toward implementing their curricula. We believe this reflected a need for additional infrastructure and organizational support to ensure project completion. To address this for cohort Two, we enhanced the mentoring structure by providing training on working with mentors and explicitly setting expectations for activities to be conducted between sessions, including regular virtual meetings. Each scholar was also assigned a near peer mentor, a recent HEALZ graduate who provided on-the-ground assistance. In addition, the group elected a team leader charged with scheduling regular meetings for the cohort to provide additional opportunities for collaboration and problem solving. To hold scholars accountable, we assigned a structured project snapshot (Appendix 3, available at https://evaaagaard1206.wixsite.com/website) for completion after each session. The snapshot guides the scholars through the tasks to be completed between weeks and is sent to both the program leadership and the mentoring team for feedback.

Some financial support was added to the program to enable scholars to employ a research assistant. In addition, the committee reviewing human subjects proposals was briefed on the nature of HEALZ projects, which may facilitate the approval process. The issue of protected time for scholars was not able to be addressed.

Faculty development in curriculum development and educational leadership are not new [11,12,13,14]. However, relatively few exist in Africa [15] and even fewer are designed as a partnership initiative specifically to meet the faculty development needs of an African country [16,17]. This is the first such program to be developed in Zimbabwe.

To ensure sustainability after grant funding concludes, we developed a train-the-trainer workshop, which was implemented in 2014 to empower interested faculty to implement ongoing faculty development in teaching, curriculum development, and program evaluation. These UZCHS faculty became core HEALZ faculty and, ultimately, took ownership of the program, which has been integrated into the new Department of Health Professions Education. In addition, HEALZ graduates were progressively incorporated into HEALZ as mentors for new scholars with the hope of developing a community of educators who are both skilled and dedicated to the mission of competency-based education. HEALZ represented only one of several aspects of the NECTAR program in Zimbabwe. Together, these programs were associated with significant gains in faculty retention, as well as student and post-graduate enrollment. Full-time faculty grew by 36% (122 to 166), annual postgraduate enrollment increased by 61% (75 to 121), and medical student enrollment increased by 70% (123 to 210). Interviews of faculty and trainees suggest that HEALZ was a significant contributor to these results (Hakim et al., “Medical Education Partnership Initiative (MEPI) in Zimbabwe: Outcomes and Challenges.” Accepted for Publication in Global Health: Science and Practice, Dec. 2017 estimated publication date) [18]. Ongoing institutional support and the involvement of the UZCHS faculty in the leadership of this program will be critical to ensure sustainability.

## Additional Files

The additional files for this article can be found as follows:

10.29024/aogh.19.s1 Appendix 1. Curriculum Overview (https://evaaagaard1206.wixsite.com/website).

10.29024/aogh.19.s2 Appendix 2. Curriculum Project Rubric (https://evaaagaard1206.wixsite.com/website).

10.29024/aogh.19.s3 Appendix 3. HEALZ Project Snapshot Week 1 (https://evaaagaard1206.wixsite.com/website).
