# Supplementary material for: Health Education Advanced Leadership for Zimbabwe (Healz): Developing the Infrastructure to Support Curriculum Reform
Source: Ann Glob Health. 2018 Apr 30;84(1):176–82. doi: 10.29024/aogh.19 (PMC6748278; doi:10.29024/aogh.19)
Supplement: Appendix 1. — Curriculum Overview (https://evaaagaard1206.wixsite.com/website). [file agh-84-1-19-s1.pdf]

Module 1

Day	Time	Session	Objectives<br>At the completion of this session, scholars will be able to:	Assigned Reading	Materials
Mon	8:30 – 10:00<br><br>(1.5 hour)<br><br>(Tea Break)	Orientation & Goal Setting: Setting a Destination and Charting Your Course	1. Describe the objectives and expectations of the HEALZ program<br>2. Reflect on personal career goals<br>3. Explain how HEALZ may help achieve those career goals	Castiglioni et al. *Succeeding as a Clinician Educator*	Orientation & Goal Setting PPT<br><br>Goal setting worksheet
	10:30 – 11:30<br><br>(1 hour)	Panel: Tips for Success from HEALZ Cohort 1 Scholars	1. Identify key elements to achieve success as a HEALZ scholar<br>2. Describe common challenges scholars may face and potential means to overcome them		HEALZ Panel Guide (a Word doc with questions for panelists)
	11:30 – 13:00<br><br>(1.5 hour)	Being an effective mentee	1. Recognize the importance of mentoring for your success<br>2. Describe the responsibilities of the mentor<br>3. Describe the responsibilities of the mentee<br>4. Develop strategies to prevent and address common reasons why mentoring relationships fail<br>5. Consider who might be an effective mentor for your HEALZ project	Straus et al. *Characteristics of Successful & Failed Mentoring Relationships*<br><br>Zerzan, et al. *Making the Most of Mentoring*	Being an Effective Mentee PPT

**Tues**	8:30 – 10:00 (Tea Break) 10:30 – 13:00<br><br>(4 hours)	Principles of Competency-Based Curriculum Development and Evaluation	1. Define "curriculum"<br>2. List the 6 steps of the Kern model and apply these to basic educational examples<br>3. Describe a framework for curriculum and program evaluation<br>4. Begin to develop ideas for a curricular project for HEALZ	Kern Chapter 1	Curriculum Development_Overview PPT<br><br>Handouts:<br>• Curriculum Development Overview Handout<br>• Learning Objectives Taxonomy<br>• Sample Verbs for Learning Objectives
**Wed**	8:30 – 10:00 (Teak Break) 10:30 – 12:00<br><br>(3 hours)	Performing a curricular needs assessment:<br>Global needs assessment (literature and curriculum review) and<br>Targeted needs assessment	1. Explain why a needs assessment is important<br>2. Describe the difference between a general needs assessment and a targeted needs assessment<br>3. Review methods to conduct a needs assessment and how to use the results to inform your curriculum	Kern Chapters 2 & 3;<br><br>Hauer & Quill, Educational Needs Assessment	Needs Assessment PPT<br><br>2014 Competency Results for MMeds and Yr4&5 Students (2 word docs)<br><br>HEALZ Project Snapshot Worksheet
	12:00 – 13:00<br><br>(1 hour)	Independent group work to brainstorm curriculum design topics	1. Choose whether project will be individual or within team<br>2. Develop preliminary project ideas<br>3. Identify a focus for needs assessment		
**Thur**	8:30 – 10:00 (Tea break)<br><br>(1.5	Evaluation Methods Part 1: Selection of methods and Survey Development	1. Select the appropriate evaluation method(s) to address your question and triangulate data (survey methods, qualitative methods, mixed methods, etc.)<br>2. Discuss the key features of a quality survey.<br>3. Co-develop a survey with fellow HEALZ participants on a topic common to the group	Kern Chapters 2 & 3	Survey Development PPT<br><br>Handouts:<br>• Survey Checklist<br>• Survey Items in

	hour)		4. Discuss how to enter and store data (data entry and online survey tools)		Need of Improvement
	10:30 – 13:00<br><br>(2 .5 hours)	Evaluation Methods Part 2: Qualitative Methods	1. Demonstrate how to conduct a qualitative research study (interview protocols, participant selection, confidentiality agreements, best practices in interviewing, recording, transcription, note-catchers)<br>2. Conduct a quality interview using a protocol (provide feedback to your partner about their interviewing skills; discuss record-keeping).	Kern Chapters 2 & 3<br><br>Hanson et al article: *Qualitative Methods for Medical Education*	Qualitative Data Collection PPT<br>"Good" and "Bad" interview videos<br><br>Handout:<br>• Interview Note-Catcher
**Fri**	8:30 – 10:00<br><br>(1.5 hours)	Expectations and planning for individual and team work	1. Discuss online expectations between now and Module 2 including:<br>a. Identification of local and distance mentors<br>b. Completion of needs assessment<br>c. Regular discussion with mentors<br>2. Use Appreciative Inquiry to describe your strengths as an individual<br>3. Develop a team resume to explain how you will work as a micro-team to be successful in meeting the HEALZ expectations<br>4. Create a timeline for completion of activities		Expectations and Planning for Independent Work PPT (need to update dates in PPT for each cohort).<br><br>HEALZ Project Snapshot worksheet
	10:00 – 12:00	Attend previous cohort's Graduation			
	12:00 – 13:00	Individual appointments (sign-up)	1. Get individualized or group help to design your needs assessment or discuss your curricular ideas		

Module Two

Day	Time	Session	Objectives	Assigned readings	Materials
Mon	8:30-10:30<br><br>(2 hours)	Week 2 Overview and<br>Works in Progress 1<br>10 min presentation of your background literature and needs assessment results-	Assessing Progress to Date<br>Presenters: (INSERT NAMES)		Week 2 Overview PPT<br><br>Provide scholars with flip charts and markers<br><br>HEALZ Project snapshot week 2
	11-13:00<br><br>(2 hours)	Analyzing Your Results Part 1: Quantitative Methods	1. Describe common quantitative statistical methods used in medical education (descriptive statistics, disaggregating data, summarizing data, sub-scales)<br>2. Apply statistical methods to curriculum and program evaluation projects<br>3. Presenting quantitative results	Windish & Diener-West: *A Clinician-Educator's Roadmap to Choosing and Interpreting Statistical Tests*	Quantitative Analysis PPT<br><br>Quant Workbook example (excel)<br><br>Handouts:<br>• How to Install Excel Analysis ToolPak<br>• Using Excel for Analyzing Survey Questionnaire<br>• What analyses used exercise
	14:00-17:00	OPTIONAL Data Analysis Support			
Tues	8:30-10:30<br><br>(2 hours)	Works in Progress 2<br>10 min presentation of your background literature and needs assessment results	Assessing Progress to Date<br>Presenters: (INSERT NAMES)		Provide scholars with flip charts and markers
	11-13:00<br><br>(2 hours)	Analyzing Your Results Part 2: Qualitative Methods	1. Discuss when and how qualitative data is best utilized to inform curriculum and program development & evaluation<br>2. Describe common qualitative analysis methods (using low-tech	Hanson et al article: *Qualitative Methods for Medical Education*	Qualitative Methods PPT

			options, Excel, Word, and advanced software)<br>3. Apply qualitative analysis to curriculum and program evaluation projects		Demo materials (have open on computer):<br>• Example Interview Notes Spreadsheet<br>• Open-ended responses for coding<br>• Example transcript for coding_demo<br><br>Handouts:<br>• Example Transcript<br>• NECTAR Annual Survey results EXCERPT<br>• UNSORTED Open-ended Survey Responses<br>• HEALZ Project snapshot week 2
	14:00-17:00	OPTIONAL Data Analysis Support			
Wed	8:30-9:30<br><br>(1 hour)	Learning Theory - Introduction to Learning and Pedagogy	1. Describe key components of constructivist, experiential, and humanistic learning theories as applied to teaching practices<br>2. Apply learning theory applications to your proposed HEALZ project to begin the planning process	Dennick article: *Twelve Tips for Incorporating Educational Theory into Teaching Practices*<br>Braungart & Braungart: *Applying Learning Theories to Healthcare Practice*	Learning Theory PPT<br><br>Handouts:<br>• IRAT handout<br>• Twelve Tips article<br>• Dice
	9:30-10:30	Writing Goals and Objectives	1. Define "objectives" and "goals".<br>2. Be able to distinguish between objectives and goals.<br>3. Practice writing objectives and review with 2-3 colleagues	Kern Ch 4	Goals and Objectives PPT

	(1 hour)				Handouts:<br>• Writing Goals and Objectives Template Worksheet<br>• Curriculum Development Worksheet<br>• Learning Objectives Taxonomy Handout<br><br>UZCHS Competencies (optional)<br>HEALZ Project snapshot week 2
	11-13:00<br><br>(2 hours)	Educational Strategies	1. Recognize the strengths and weaknesses of various educational methods<br>2. Discuss principles of choosing an education methodology.	Kern Ch 5	Educational Strategies PPT<br><br>+ HEALZ Project snapshot week 2
Thurs	8:30-13:00<br><br>(4 hours)	Learner Assessment Strategies & Curriculum Evaluation-	1. Define and discuss various learner assessment and curriculum/ program evaluation strategies.<br>2. Apply assessment and evaluation strategies to curriculum examples from HEALZ.<br>3. Identify and define formative and summative methods for curriculum evaluation.<br>4. Apply curriculum evaluation strategies to curriculum examples from the literature.	Kern Ch 6 & 7<br><br>Norcini et. Al, Criteria for good assessment: Consensus statement and recommendations from the Ottawa 2010 Conference	Learner Assessment PPT<br><br>Flip charts<br><br>Markers<br><br>HEALZ Project snapshot week 2
	14-17:00	OPTIONAL Work Period: Curriculum Design & Evaluation			
Fri	8:30-10:30<br><br>(2 hours)	Program Evaluation	1. Identify stakeholders for your project/curriculum<br>2. Develop an evaluation plan that includes evaluation questions, methods/data sources, a timeline, and identifies who is responsible for collecting, analyzing, and reporting the results	Kern Ch 6 & 7	Program Evaluation PPT<br><br>Handouts:

					• Blank logic model<br>• Planning program evaluation worksheet<br><br>Cookies<br><br>HEALZ Project snapshot week 2
	11:00-1300<br><br>(2 hours)	Goal Setting and Team activity	1. Describe expectations for work between now and week 3<br>2. Develop a plan for achieving expectations between now and week 3<br>3. Appreciate personal and group strengths needed to achieve goals		HEALZ Project snapshot week 2

Module Three

Day	Time	Session	Objectives	Assigned Reading	Materials
		MBTI: Complete assessment online in advance of workshop			Log in information
Sun	TBD<br>(1 hour)	**Opening Session**<br>• Welcome/ ice breaker<br>• Overview of Mon-Weds schedule, expectations	Participants can describe expectations for module 3 workshops		HEALZ Intro Slides
Mon	8:30 - 10:00<br><br>(1.5 hours)	**Leading from personal strengths**	Participants can:<br>explain their MBTI type<br>describe the strengths and weaknesses of their MBTI type<br>recognize the importance of diversity of MBTI type in their teams and work environment		MTI PPT<br>• Results of online MBTI assessments<br>• MBTI books<br>• Flip chart/pens/ chairs
	10:00- 10:30	Tea break			
	10:30 - 1:00<br><br>(2.5 hours)	**Leading from understanding the strengths of others**	Participants can describe contributions of MBTI types to team success		Type Table (to be constructed on flip chart paper by Walter- see MBTI book for example)
	1:00 - 2:00	Lunch break			
	2:00 - 5:00 with informal tea break<br><br>(3 hours)	**Negotiating conflict**	Participants can describe their conflict style preferences<br>can describe the strengths and weaknesses of the various conflict styles<br>discuss personal sources of conflict and strategies that may be more or less effective in solving that conflict		Conflict Styles PPT<br><br>TKI
Tues	8:30 - 8:45	**Warm up**			LCD projector

	8:45 - 10:00	**Evaluating your curriculum**	Participants can prepare an evaluation plan for their curriculum<br>Participants can identify ways to use learner assessment results for curriculum evaluation		Evaluation PPT<br><br>Handout: Planning program evaluation worksheet
	10:00 - 10:30	Tea break			
	10:30 - 1:00	**Managing Change**	Participants identify at least two strategies to gain support for changes needed to implement their HEALZ project		Index cards with change emotions (create a digital version?)<br><br>DVD player, screen, speakers, "Our Iceberg is Melting" DVD<br><br>Handout - "What can I do?"
	1:00 - 2:00	Lunch break			
	2:00 - 3:00?	**Getting Feedback on HEALZ Curriculum Project**<br>Overview<br>• Abstract assignment<br>• July expectations: Poster template and revised poster rubric<br>How to give feedback & rubric	Participants will prepare an abstract for their HEALZ project		Poster template and revised rubric<br><br>Feedback rubric<br><br>Printer available for abstracts to be printed
	3:00 - 5:00<br>& informal tea break	Consultation/work time to prepare abstract			

Wed	8:30 - 10:00	**Getting and receiving feedback**<br>Overview of how to give effective feedback	Participants will be able to describe strategies for effective feedback		
	10:00 - 10:30	Tea break			
	10:30 - 12:00	**Getting and receiving feedback**<br>Read and provide feedback using rubric to colleagues; provide oral feedback back as well as completed rubric	Participants will get and give feedback on their abstract in groups of 3-4		Two printed copies of each person's abstract<br><br>Groups for reviewing
	12:00 - 1:00	**Closing activities**<br>Write a letter to yourself outlining leadership goals<br>Evaluation surveys	Participants will set personal leadership goals		Envelopes<br>Blank paper<br>Exit surveys
		**Lunch**			
