# Supplementary material for: Health Education Advanced Leadership for Zimbabwe (Healz): Developing the Infrastructure to Support Curriculum Reform
Source: Ann Glob Health. 2018 Apr 30;84(1):176–82. doi: 10.29024/aogh.19 (PMC6748278; doi:10.29024/aogh.19)
Supplement: Appendix 2. — Curriculum Project Rubric (https://evaaagaard1206.wixsite.com/website). [file agh-84-1-19-s2.pdf]

HEALZ Curriculum Project Rubric

HEALZ Scholars: Use this rubric to guide the development of your curriculum project. Your completed project should include all of these elements. However, your poster itself will be scored by reviewers using the Poster Rubric.

Domain	Poor	Fair	Good	Excellent
**Evidence-based**	No literature to support value and poor argument	No literature but reasonable argument to suggest value	Preliminary literature/ pilot data supports value	Body of literature/ program evaluation suggest this is a best practice
**Important/ Relevant to Student learning**	Does not address an issue of importance; no or negative impact anticipated	Minor importance to student outcomes; limited impact anticipated	Important to our student competencies; Moderate positive impact anticipated	Significant issue with major positive impact anticipated
**Important/ Relevant to Institutional Goals, status, resources**	Does not address an issue of importance; no or negative impact anticipated	Minor importance to institution; limited impact anticipated	Important to institution; Moderate positive impact anticipated	Significant issue with major positive impact anticipated (e.g, increases cross disciplinary involvement, valued by departments, broad benefit)
**Quality of stated Program Goals & Objectives**	Absent / not Documented	Poorly defined or unclear	Some clarity and definition but improvements needed	Clear and well defined
**Pedagogically Sound**	Inappropriately timed; ignores or neglects existing structures or curriculum	Ineffectively timed; minimal integration with existing structures or curriculum	Appropriately timed with clear efforts at integration into existing curriculum	Effective timing and integration; maximizing effectiveness for student learning
**Educational Strategies**	Inappropriate or ineffective educational strategy for goals/ objectives	Appropriate but minimally effective educational strategy to achieve goals/ objectives	Appropriate and effective educational strategy to achieve goals/ objectives	Uses multiple appropriate and effective educational strategies to achieve goals/ objectives
**Appropriate Learner Assessment**	No learner assessment strategy	Learner assessment strategy does not clearly track to goals/ objectives	Learner assessment strategy tracks to goals/ objectives; limited reliability or validity	Reliable and valid learner assessment strategies that track to goals and objectives
**Program Evaluation**	No program evaluation strategy	Limited program evaluation; may be difficult to determine effectiveness	Appropriate program evaluation strategy	Program evaluation strategy likely to result in scholarship/ publication
