# Supplementary material for: Health Education Advanced Leadership for Zimbabwe (Healz): Developing the Infrastructure to Support Curriculum Reform
Source: Ann Glob Health. 2018 Apr 30;84(1):176–82. doi: 10.29024/aogh.19 (PMC6748278; doi:10.29024/aogh.19)
Supplement: Appendix 3. — HEALZ Project Snapshot Week 1 (https://evaaagaard1206.wixsite.com/website). [file agh-84-1-19-s3.pdf]

Proposal Snapshot Template

Project Title:

1. **What do you think is an important curricular problem or issue?**

2. **Why do you think your curriculum problem/issue is important? How do you think your project will improve/impact student (or other level learner) learning?**

3. **How will you prove that your problem is important?**
    a. What databases/resources will you use to search for other research/ or projects on your topic?

    b. What resources can you call upon for assistance locally? Elsewhere?

    c. How will you organize the citations you discover?

    d. Key notes/ important things specific to your project:

4. **How will you know that a gap really exists? Which existing data can gather? Which local stakeholders or groups might you need to engage in this process? What do you need to know from them?**

5. **What methods will you use to gain their input/ opinion?**

6. **What kind of analysis will you do? Will you use qualitative analysis methods? Quantitative/ statistical methods? Will you need help with this analysis?**

    a. Do you need more instruction? If so, in what area?

    b. Do you want to have a statistician/ qualitative researcher do the analysis for you? Are there resources for this?

7. **Who will you work with to accomplish your goals over the next few months? Who will do what? How will you communicate? How will you hold each other accountable?**

8. **How will you make your work public?** (begin thinking about this now)

9. **What questions still remain?**

10. **What follow-up research or project do you hope to do based on this work?**
